# Correlation between FFP transfusion and age in ICU patients

**DOI:** 10.1186/2197-425X-3-S1-A379

**Published:** 2015-10-01

**Authors:** A Vakalos, V Popko

**Affiliations:** ICU, Xanthi General Hospital, Xanthi, Greece

## Introduction

While plasma donation is still necessary as a unique source of human proteins and to treat coagulation disorders, FFP administration seems to have high rate of inappropriate indication. After all, FFP transfusion is not risk free, and is associated with lung injury, infectious disease and circulatory overload in recipients. On the other hand, elderly patients may have impaired physical status and increased demand for FFP transfusion.

## Objectives

The aim of our retrospective observation study was to test the hypothesis that a correlation exists between FFP transfusion and age, in our both medical and surgical ICU served in community hospital.

## Methods

From January 2006 to June 2014 admitted to our ICU 620 patients, mean age 64.8 years, mean length of ICU stay (LOS) 14.2 days, mean mechanical ventilation duration per ventilated patient (V. Days) 12.23 days, mean APACHE II score on admission 21.2, predicted mortality 38.9%, actual mortality 31.45%, Standardized Mortality Ratio (SMR) 0.80. From our database we looked for age and the following values and indexes according FFP transfusion per year from 2006 to 2014 (mean values). Total, per patient, per hospitalization days (HD), per patient under mechanical ventilation (pts V) and per ventilation days (VD) Using linear correlation method, we looked for linear slope, correlation coefficient (r), and coefficient of determination (r^2^), and by linear regression method using ANOVA test we looked for p value, according age and FFP transfusion.

## Results

**Table 1:** Correlation between age and FFP transfusion index

FFP	Slope	r	r2	S. Error	Lower C.I.	Upper C.I.	p value
Total	1.807	0.2794	0.0780	2.348	-3.745	7.36	0.4666
Per patient	-0.002	-0.018	0.000	0.041	-0.099	0.095	0.9621
Per Hosp. Day	-0.002	-0.301	0.0906	0.003	-0.009	0.004	0.4311
Per pt ventilated	-0.007	-0.066	0.0044	0.043	-0.110	0.095	0.8640
Per Vent. Day	-0.016	-0.144	0.0208	0.004	-0.011	0.008	0.7107

## Conclusions

According to our data, there was no statistically significant correlation detected between age and FFP transfusion indexes. Our data suggest that even though some older patients may need more FFP than younger, FFP transfused do not correlate statistically significant with age in ICU patients.

